# Self-Expanding Metallic Stent Fracture in the Treatment of Malignant Biliary Obstruction

**DOI:** 10.1155/2018/6527879

**Published:** 2018-04-11

**Authors:** Chuanguo Zhou, Baojie Wei, Jianfeng Wang, Qiang Huang, Hui Li, Kun Gao

**Affiliations:** Department of Interventional Radiology, Beijing Chaoyang Hospital, Affiliated Hospital of Capital Medical University, Beijing 100020, China

## Abstract

**Background:**

Palliative therapies for malignant biliary obstruction (MBO) include choledochojejunostomy and self-expanding metallic stent (SEMS) insertion. Fractures following SEMS insertion in MBO treatment are scarce.

**Objective:**

To assess the clinical features of biliary stent fractures and evaluate associated factors.

**Methods:**

One hundred fifty-six consecutive patients who underwent biliary SEMS placement for MBO treatment at Beijing Chaoyang Hospital affiliated to Capital Medical University, in 2010–2015, were evaluated retrospectively. Demographics, clinical features, stent parameters and patency times, and survival times were collected. Across the ampulla of Vater, balloon dilatation, number of stents, stent patency time, and survival time were compared between the stent and nonstent fracture groups.

**Results:**

There were 168 biliary metallic stents inserted in 156 patients, including 144 and 12 patients with one and 2-3 stents, respectively. Pre- and/or postballoon dilation was performed in 107 patients. Stents across and above the duodenal papilla were used in 105 and 51 patients, respectively. Six cases (3.8%) with stent occlusion had stent fractures. Single- and multiple-stent fracture rates were 4/144 (2.8%) and 2/12 (16.7%), respectively. Fracture times after stent deployment were 126.8 ± 79.0 (median, 115.5) days. Stent patency times in the stent and nonstent fracture groups were 151.8 ± 67.8 (median, 160.5) days and 159.3 ± 73.6 (median, 165.5) days, respectively. Overall survival times in the stent and nonstent fracture groups were 399.7 ± 147.6 (median, 364.0) days and 283.7 ± 126.1 (median, 289.0) days, respectively.

**Conclusion:**

Stent fractures following MBO treatment constitute a relatively rare long-term complication. Though there were no factors found to be significantly associated with SEMSs fracture, a trend could be observed towards more fractures in multistent, transpapillary, and balloon dilation groups.

## 1. Introduction

Malignant biliary obstruction (MBO) refers to the blockage of the biliary tree by direct malignant tumor infiltration or external compression; the underlying malignancies in the vast majority of cases are cholangiocarcinomas and pancreatic head carcinomas. Other tumors in this region include cystic, duodenal, and gastric cancers, as well as intrahepatic and hilar lymph node metastases [1]. Commonly, the clinical manifestation of MBO is painless jaundice, with anorexia and weight loss. Infection secondary to malignant bile duct obstruction may result in cholangitis, symptoms of the digestive tract, hepatic failure and renal insufficiency, sepsis, and even death. Despite recent advances in radiology, only approximately 20% of periampullary tumors are resectable at the time of diagnosis, because of invasiveness, metastasis, and poor performance status [2]. The available options for palliative therapy of malignant biliary tree obstruction include choledochojejunostomy, percutaneous external/internal drainage or metallic stent placement, and endoscopic techniques. Recent evidence indicates the superiority of self-expanding metallic stents (SEMSs) over plastic stents, with regard to stent patency time, complications rates, and reinterventions. In selected subgroups of patients, SEMS placement may have survival benefits [3]. Short-term complications of SEMS insertion include cholangitis, asymptomatic increase of amylase levels, and bleeding. Long-term complications comprise stent occlusion due to tumor ingrowth or overgrowth, stent migration, and viscus perforation [4]. Few reports have described stent fractures following SEMS insertion in the treatment of malignant biliary obstruction [5–11]. The present study aimed at assessing the clinical features of biliary stent fractures in six cases and identifying factors that may affect biliary stent fractures.

## 2. Materials and Methods

The study was reviewed and approved by the ethics committees of Beijing Chaoyang Hospital affiliated to Capital Medical University according to the standards of the Declaration of Helsinki. Written informed consent was waived because of the retrospective nature of this study. All patient records were anonymized and deidentified before analysis.

A total of 156 consecutive patients who underwent biliary SEMS placement with percutaneous transhepatic approach for the treatment of MBO at Beijing Chaoyang Hospital affiliated to Capital Medical University between January 2010 and September 2015 and were not suitable for surgery or declined resection for poor performance status were enrolled in this retrospective study. Most of the patients with malignant biliary obstruction were accompanied by cholangitis. To the patients which have no cholangitis but with obvious gastrointestinal symptoms accompanied by intrahepatic biliary dilatation and serum total bilirubin above 150 *μ*mol/L, they were also treated by biliary drainage and stent implantation. Obstruction location, stent type, number of stents placed, placement across the duodenal ampulla, and predilation or postdilation were recorded. If applicable, stent fracture data were extracted from patient records and relevant imaging findings. Malignant biliary obstructions include “distal” and “proximal” obstruction types. Distal biliary obstruction refers to an obstruction below the main hepatic ducts' confluence. In such cases, the entire biliary tree could be drained with a single stent without requiring an external catheter.

## 3. Interventional Procedure

According to preoperative computed tomography scan (CT) or magnetic resonance imaging (MRI) data, the optimal puncture point in most cases was 2–4 cm below the right cardiophrenic angle at the axillary midline. A 22-gauge double-walled needle, for example, the Chiba needle, was advanced under fluoroscopic guidance. The stylet was removed, and the contrast agent was gently injected while retracting the needle until opacification of the bile duct. Once the target bile duct was accessed, a 0.018^″^ wire was advanced into the duct through a coaxial system, and the wire was upsized to a 0.035^″^ guide wire. A 7.0–8.5 French biliary drainage catheter (COOK Inc., Bloomington, IN, United States) was inserted into the dilated biliary duct under wire guidance. Two to four weeks later, serum total bilirubin levels were decreased to almost normal amounts, and cholangitis was controlled, with the patient's general condition improved. Then, the uncovered nitinol SEMSs (COOK Inc., Bloomington, IN, United States) were implanted. Through an 8F sheath, a directional catheter in combination with a hydrophilic guide wire was used to cross the biliary obstruction and advanced into the small bowel. An appropriately sized stent may be inserted, usually 8 or 10 mm in diameter and 40 to 100 mm in length. According to the degree of biliary stenosis and stent expansion, pre- or postdilation with balloon catheter was selected. Cholangiography via the sheath after stent deployment was used to assess stent patency. In case of satisfactory contrast agent flow, the sheath was removed, with the puncture tract plugged with gel foam pledges. Stent fracture was defined as persistent or recurrent jaundice, and cholangiography revealed bile duct obstruction, with interruption of biliary stent continuity or deficiency of the biliary stent segment.

## 4. Perioperative Treatment

All patients received 150 mg of magnesium isoglycyrrhizinate, 40 mg of pantoprazole, and 6 g (three doses) of cefoxitin sodium intravenously guttae at the day of the procedure. Continuous intravenous infusion of octreotide 0.3 mg was administered before procedure to prevent acute pancreatitis. Postoperative serum amylase levels were used to determine whether to continue intravenous octreotide administration. Nausea, vomiting, abdominal pain, and other symptoms were treated as well. The patients were followed up via outpatient appointments or by telephone interviews every 3 months to 1 year after the procedure. The last follow-up occurred in May 2017.

## 5. Statistical Analysis

Statistical analyses were performed with the SPSS 22.0 software (SPSS, United States). Quantitative data are expressed as mean ± SD and were compared by unpaired *t*-test; categorical variables were compared by the Chi-square test or Fisher's exact test. Stent patency time and survival time were derived by the Kaplan–Meier method and compared using the log-rank test. Two-tailed *P* < 0.05 was considered statistically significant.

## 6. Results

A total of 156 patients were treated during the study period, including 94 men (65.6 ± 11.4 years old) and 62 women (68.6 ± 12.0 years old). Biliary obstructions in these patients were pancreatic cancer (*n* = 72), cholangiocarcinoma (*n* = 40), malignant gastric carcinoma (*n* = 21), cancer of the ampulla of Vater (*n* = 10), gallbladder carcinoma (*n* = 7), colon cancer metastasis (*n* = 3), lung cancer metastasis (*n* = 2), and ovary cancer metastasis (*n* = 1). The success rate of the biliary stent procedure was 100%. A successful procedure consists of gastrointestinal symptoms relieved, total bilirubin decreased to almost normal, and biliary stent well positioned and patent. All patients had “distal” biliary obstruction. The most common short-term complications included cholangitis, asymptomatic amylase increase, and biliary bleeding. Long-term complications comprise stent occlusion due to tumor ingrowth or overgrowth and stent fractures. There were no patients who had stent migration, viscus perforation, or seeding (Table 1).

**Figure 1:**
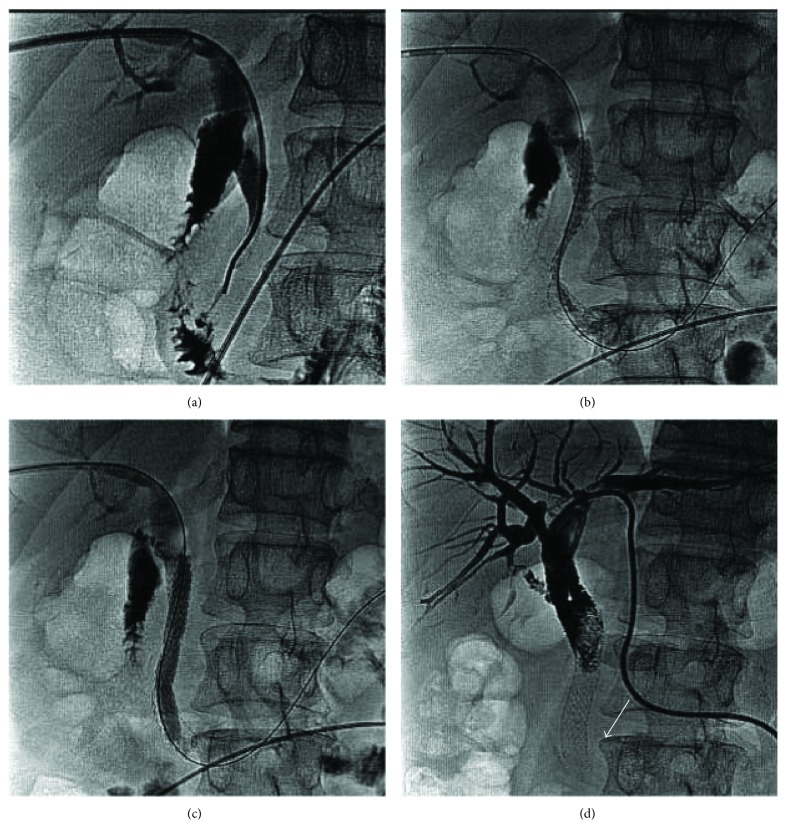
A 62-year-old male patient with pancreatic carcinoma whose common bile duct was severely obstructed by compression from pancreatic head carcinoma. (a) Contrast medium injection via the percutaneous transhepatic approach showing the distal common bile duct severely obstructed. (b) Five weeks later, a biliary stent (diameter, 10 mm; length, 80 mm; COOK Inc., Bloomington, IN, United States) was deployed in the distal common bile duct. The guidewire can be observed in the distal duodenum. The distal part of the stent protrudes into the duodenum. (c) Because of incomplete dilation of the biliary stent, a balloon (diameter, 8 mm; length, 40 mm; ATB5-35-40-8-4.0; COOK Inc.) was used for biliary-plasty. (d) Eighty-nine days after stent placement, a left percutaneous transhepatic drainage catheter was placed for recurrent biliary obstruction. Cholangiography showed the biliary stent completely fractured (arrow) at the level of the stricture.

**Figure 2:**
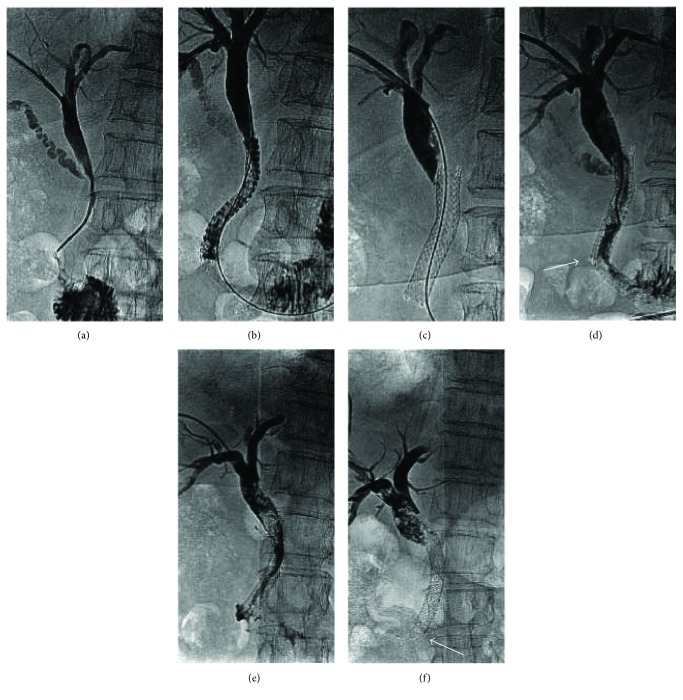
Complete stent fracture in an 81-year-old woman with common bile duct obstruction secondary to a cystic mass of the pancreatic head. (a). Percutaneous transhepatic cholangiogram depicting common bile duct stricture was shown. (b). Six weeks later, predilation (diameter, 8 mm; length, 40 mm; ATB5-35-40-8-4.0; COOK Inc.) was performed and a stent (diameter, 8 mm; length, 60 mm; COOK Inc., Bloomington, IN, United States) was inserted in the stricture, extending from the duodenum to the cystic duct. (c, d). Eleven months after biliary stent placement, the patient developed reobstructive jaundice, and percutaneous cholangiography showed stent occlusion. Two additional stents (diameter, 8 mm; length, 60 mm; COOK Inc., Bloomington, IN, United States) were deployed. An obtuse angle was formed distally between the two biliary stents (arrow). (e, f). Thirty-seven days after implantation of the two biliary stents, plain X-ray revealed the absence of the distal part of the stent (arrow), resulting from complete stent fracture. The fractured stent had therefore passed through the bowel without incident.

**Figure 3:**
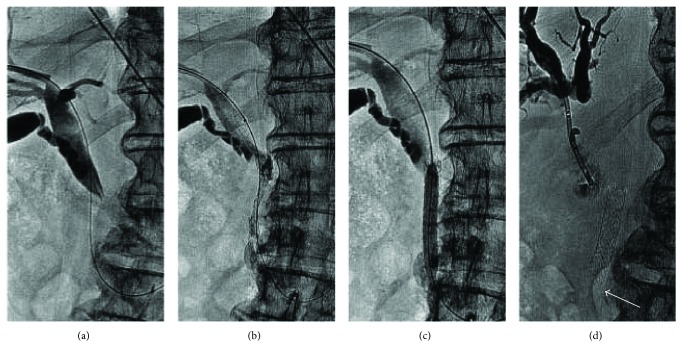
Complete stent fracture in a 79-year-old man with common biliary obstruction from pancreatic cancer. (a). Percutaneous transhepatic cholangiogram demonstrating severe stenosis of the distal common bile duct was shown. (b). After biliary decompression for 5 weeks, a metallic, self-expandable stent (diameter, 10 mm; length, 60 mm; COOK Inc., Bloomington, IN, United States) was inserted into the distal common bile duct. (c). Because of complete dilation of the biliary stent, a balloon (diameter, 8 mm; length, 40 mm; ATB5-35-40-8-4.0; COOK Inc.) was used for biliary-plasty. (d). Sixty-five days after stent deployment, percutaneous transhepatic cholangiography revealed biliary obstruction. The distal part of the stent was absent as a result of fracture (arrow). The fractured stent had therefore passed through the bowel without incident.

**Figure 4:**
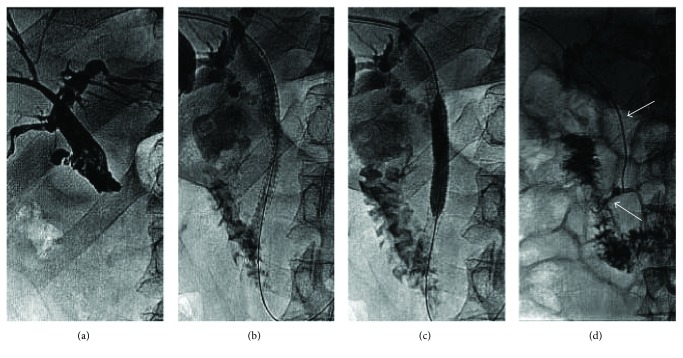
Complete stent fractures at two locations in an 82-year-old man with pancreatic cancer. (a). Percutaneous transhepatic cholangiogram demonstrating a stricture in the distal common bile duct was shown. Biliary decompression was performed with placement of a drainage catheter by the routine right approach. (b, c). Eight weeks later, a metallic self-expandable stent (diameter, 8 mm; length, 80 mm; COOK Inc., Bloomington, IN, United States) was inserted. Contrast medium injection after stent placement revealed stent patency. The distal part of the stent protruded into the duodenum. Postdilation was performed. (d). After 179 days, the patient developed recurrent jaundice. Percutaneous transhepatic cholangiography showed stent occlusion. There were two complete fracture sites in the metallic biliary stent (arrow), located at the upper biliary stenosis and duodenal ampulla, respectively.

**Figure 5:**
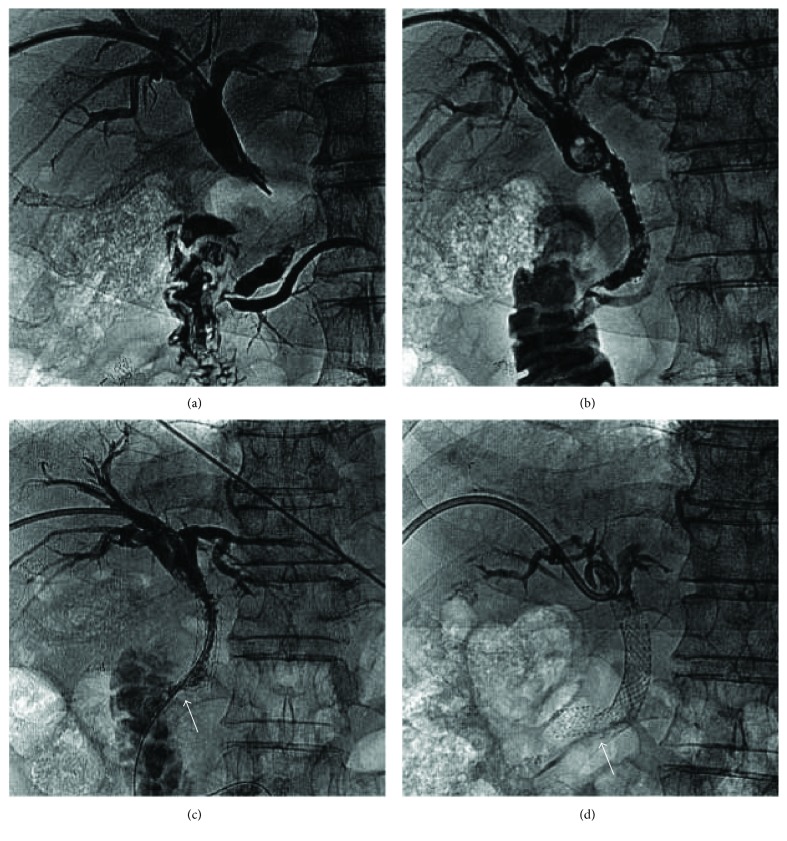
Fracture in a 71-year-old man with common bile duct obstruction secondary to cholangiocarcinoma. (a). Percutaneous transhepatic cholangiogram depicting common bile duct stricture was shown. (b). Two weeks later, a stent (diameter, 10 mm; length, 40 mm; COOK Inc., Bloomington, IN, United States) was inserted into the biliary stricture, above the duodenal ampulla. (c). Nine months after stent placement, the patient developed reobstructive jaundice, and percutaneous cholangiography revealed stent occlusion. Another biliary stent (diameter, 10 mm; length, 100 mm; COOK Inc., Bloomington, IN, United States) was deployed, extending from the duodenum to the convergence of the left and right hepatic ducts. An obtuse angle was formed between the two biliary stents (arrow). (d). One hundred and forty-two days after the second biliary stent implantation, plain X-ray showed the stent fracture located at the duodenal ampulla (arrow).

**Figure 6:**
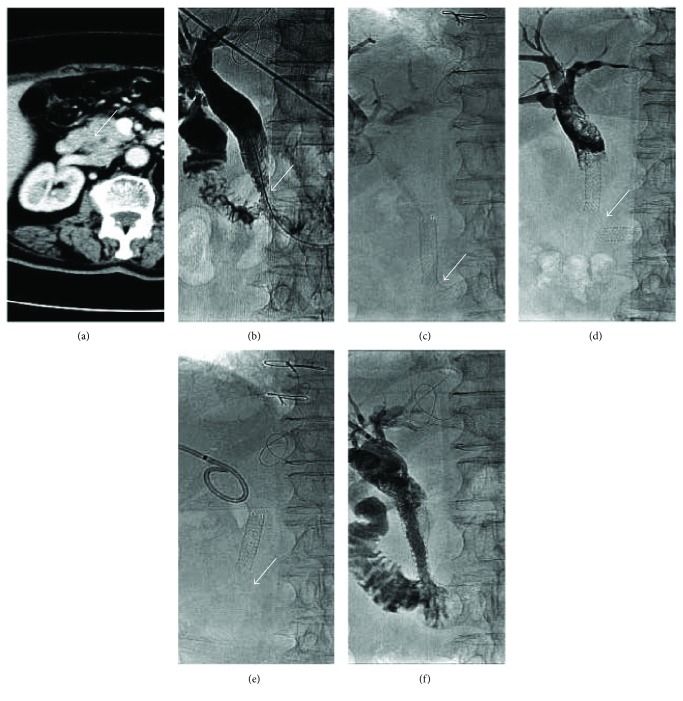
Gradual fracture of a biliary stent in a 78-year-old woman with common bile duct obstruction secondary to carcinoma of the ampulla of Vater. (a). Abdominal-enhanced CT scan showing a mass located at the duodenal ampulla of Vater (arrow) was shown. (b). Percutaneous transhepatic cholangiogram showing severe stricture of the distal common bile duct was shown, and a SEMS (diameter, 8 mm; length, 60 mm; COOK Inc., Bloomington, IN, United States) was inserted to relieve the obstruction. (c)–(e). 249 days after SEMS insertion, percutaneous transhepatic cholangiography showed stent fracture located at the stricture position, from partial, filamentous connective to complete fracture, without the distal part of the stent (arrow). (f). A new SEMS (diameter, 8 mm; length, 80 mm; COOK Inc., Bloomington, IN, United States) was inserted to relieve the reobstruction.

There were 168 biliary metallic stents inserted in 156 patients, including 144 and 12 (7.7%) patients with one and 2-3 stents, respectively. Pre- and/or postballoon dilation was performed in 107 patients; the remaining patients did not undergo balloon dilation. Stent across and above the duodenal papilla was adopted in 105 and 51 patients, respectively. Six cases (3.8%) with stent occlusion had stent fractures. Single- and multistent fracture rates were 4/144 (2.8%) and 2/12 (16.7%), respectively. Mean and median fracture times after stent deployment were 126.8 ± 79.0 days and 115.5 days, respectively. Meanwhile, stent patency times in the stent and nonstent fracture groups were 151.8 ± 67.8 (median, 160.5) days and 159.3 ± 73.6 (median, 165.5) days, respectively. Overall survival times in the stent and nonstent fracture groups were 399.7 ± 147.6 (median, 364.0) days and 283.7 ± 126.1 (median, 289.0) days, respectively. The characteristics of these six patients are summarized in Tables 2 and 3 and Figures 1–6.

## 7. Discussion

Biliary stent placement is widely used in the treatment of a variety of malignant biliary obstruction cases. For patients with malignant biliary obstruction and life expectancy exceeding 3 months, self-expandable metal stent deployment is the standard treatment option [12, 13]. There are two techniques for biliary stent insertion, including endoscopic and percutaneous hepatic approaches; nevertheless, there has not been a uniform conclusion published on either the efficacy of the two types of drainage or the incidence rate of complications. The procedure's success rate is nearly 100% [5].

Common short and long-term complications include cholangitis, asymptomatic increase of amylase, and occlusion due to tumor ingrowth or overgrowth [14, 15]. To the best of our knowledge, only 7 reports have described a total of 13 patients with fractures of metallic biliary stents placed to relieve malignant biliary obstruction (Table 4) [5–11]. The incidence of biliary stent fracture is approximately 7–22% [7, 9]; in this study, it was 3.8%. Biliary reobstruction is expected from disease progression, but stent failure due to fracture is usually not suspected and thus probably overlooked and underreported. Therefore, it is difficult to estimate the exact incidence rate of biliary stent fractures.

Nitinol is an alloy consisting of 55% nickel and 45% titanium. Its biocompatibility as well as unusual and useful properties of shape memory explains its widespread use in medicine. Stents may be exposed to significant stress-induced fatigue, which may weaken the metallic structure over time, leading to subsequent fracture and fragmentation [16]. Nitinol SEMSs are widely used in the management of stenosis affecting vascular and nonvascular vessels, such as the coronary artery, lower extremity artery, subclavian vein, and esophagus [16–21]. The most common factors which may affect stent fractures may include balloon dilation or stent overexpansion, stent overlap, stent length, stent type and stent conformability, and tortuous and highly angulated vessels [13, 22]. The anatomic sites of biliary stent fractures varied; most were located at overlapping stents, tumor sites, or the ampulla, with distal stent disconnection. This may be related to increased cutting force, which caused significant stress-induced fatigue and resulted in fracture. The possibility of disease- or treatment-related factors leading to stent fracture has been considered.

The present study showed that the multistent fracture rate (16.7%) was higher than the single stent counterparts (2.8%) (*P* = 0.07). The differences not reaching statistical significance may be due to the small sample size. The interactions between the stents and stent geometry during multistent implantation are considered the most important factor in biliary obstruction fractures. In addition, duodenal peristalsis and tumor compression can promote stent fracture occurrence. Operation-related factors, such as selecting stent size, passing the stent across the ampulla (*P* = 0.09), and pre- or postdilation (*P* = 0.10), may also contribute to stent fracture development [17]. Concerning stent patency time, no significant difference between the fracture and nonfracture groups was obtained (*P* = 0.52). As shown above, the survival time difference (*P* = 0.16) may be related to the advanced age of patients, low-grade malignancy, and the small sample size. Treatment of biliary stent fractures includes reimplantation of the biliary stent, indwelling of a new biliary drainage catheter, and partial removal of the fractured stent by endoscopy.

In conclusion, stent fracture occurs after insertion of self-expanding nitinol stents for the treatment of malignant biliary obstruction, as a relatively rare long-term complication. The reported low incidence of this complication may be due to lack of awareness about stent fractures and the difficulty in detecting them. Though there were no factors found to be significantly associated with SEMSs fracture, a trend could be observed towards more fractures in multistent, transpapillary, and balloon dilation groups.

## Figures and Tables

**Table 1:** The most common short- and long-term complications of SEMS deployment in the 156 patients.

	Type of complications	Patients
Short-term complications	Cholangitis	33 (21.2%)
	Asymptomatic amylase increase	21 (13.5%)
	Biliary bleeding	8 (5.1%)
Long-term complications	Ingrowth	86 (55.1%)
	Overgrowth	43 (27.6%)
	Stent fractures	6 (3.8%)

**Table 2:** Demographic characteristics, across/above the ampulla, balloon dilation, number of stents, stent patency time, and survival time of the 156 study patients.

	Nonstent fracture (*n* = 150)	Stent fracture (*n* = 6)	*t*/*χ*^2^	*P* value^※^
Sex, M/F	90/60	4/2	0.107	0.55
Mean age ± SD, years	67.1 ± 11.6	76.0 ± 8.0	1.852	0.07
Above the ampulla, *n*	51	0	3.031	0.09
Across the ampulla, *n*	99	6		
Nondilation, *n*	49	0	2.858	0.10
Balloon dilation, *n*	101	6		
Single stent, *n*	140	4	5.778	0.07
Multistent, *n*	10	2		
Stent patency time, days	159.3 ± 73.6 (median, 165.5)	151.8 ± 67.8 (median, 160.5)	0.414	0.52
Survival time, days	283.7 ± 126.1 (median, 289.0)	399.7 ± 147.6 (median, 364.0)	5.952	0.16

SD: standard deviation, ^※^Nonstent fracture versus stent fracture groups.

**Table 3:** Clinical characteristics of the six patients.

Patient	Case 1	Case 2	Case 3	Case 4	Case 5	Case 6
Age, gender	62, male	81, female	82, male	82, male	71, male	78, female
Primary disease	Pancreatic carcinoma	Pancreatic carcinoma	Pancreatic carcinoma	Pancreatic carcinoma	Cholangiocarcinoma	Duodenal ampulla carcinoma
Level of obstruction	Distal	Distal	Distal	Distal	Distal	Distal
Parameters of stent	10 mm × 80 mm^※^	First stent, 8 mm × 60 mm; next two stents, 8 mm × 60 mm	10 mm × 60 mm	8 mm × 80 mm	First stent, 10 mm × 40 mm; second stent, 10 mm × 100 mm	8 mm × 60 mm
Number of stents	One	Three	One	One	Two	One
Pre-/postdilation	Yes	Yes	Yes	Yes	Yes	Yes
Across the ampulla	Yes	Yes	Yes	Yes	Yes	Yes
Time to stent fracture (days)^※※^	89	37	65	179	142	249
Patency time of the stent (days)	89	187	65	179	142	249
Overall survival time (days)	241	312	335	393	662	455

^※^10 mm is the stent diameter and 80 mm the length. ^※※^Time to stent fracture is defined as the time period from last stent insertion to stent fracture.

**Table 4:** Previous reports assessing metallic biliary stent fractures.

Authors (years)	Patients	Primary disease	Time to fracture	Number of stents	Type of stent	Treatment of stent fracture
Peck and Wattam [7]	*n* = 4/66^※^ Female (*n* = 3) Male (*n* = 1) Age, 78.5 ± 5.6 years	Pancreatic carcinoma (*n* = 2); cholangiocarcinoma (*n* = 1); recurrent cholangitis (*n* = 1)	Median fracture times, 225 days	One stent in each patient	Nitinol stent	Reimplantation
Yoshida et al. [5]	82, male	Pancreatic carcinoma	5 months	One	Nitinol stent	Reimplantation
Sriram et al. [10]	63, female	Cholangiocarcinoma	6 months	One	Nitinol stent	Reimplantation
Yoshida et al. [6]	82, male	Cholangiocarcinoma	18 months	Two	Nitinol stent	Reimplantation
Rasmussen et al. [9]	*n* = 4/48 Female (*n* = 3) Male (*n* = 1) Age, 71.3 ± 13.8 years	Cancer of the papilla of Vater (*n* = 1); pancreatic (*n* = 3)	Median fracture times, 392 days	One stent in each patient	Nitinol stent	Reimplantation
Saravanan et al. [8]	50, male	Benign biliary anastomotic stricture	NA	One	Nitinol stent	The surgical intervention
Alkhiari et al. [11]	67, female	Recurrent cholangitis	NA	One	Nitinol stent	Remove the fractured stent by endoscopy

^※^Biliary stent fractures occurred in 4/66 patients after biliary stent implantation.
